# Comparison of RISK-PCI, GRACE, TIMI risk scores for prediction of major adverse cardiac events in patients with acute coronary syndrome

**DOI:** 10.3325/cmj.2017.58.406

**Published:** 2017-12

**Authors:** Tamara Jakimov, Igor Mrdović, Branka Filipović, Marija Zdravković, Aleksandra Djoković, Saša Hinić, Nataša Milić, Branislav Filipović

**Affiliations:** 1 Department of Cardiology, Clinical and Hospital Center “Bežanijska Kosa”, Belgrade, Serbia; 2 Emergency Center, Clinical Center of Serbia and Faculty of Medicine, University of Belgrade, Belgrade, Serbia; 3 Department of Gastroenterology, Clinical and Hospital Center “Bežanijska kosa” and Faculty of Medicine, University of Belgrade, Belgrade, Serbia; 4 Department of Cardiology, Clinical and Hospital Center “Bežanijska kosa” and Faculty of Medicine, University of Belgrade, Belgrade, Serbia; 5 Institute for Medical Statistics and Faculty of Medicine, University of Belgrade, Belgrade, Serbia; 6 Institute of Anatomy “Niko Miljanić” and Faculty of Medicine, University of Belgrade, Belgrade, Serbia

## Abstract

**Aim:**

To compare the prognostic performance of three major risk scoring systems including global registry for acute coronary events (GRACE), thrombolysis in myocardial infarction (TIMI), and prediction of 30-day major adverse cardiovascular events after primary percutaneous coronary intervention (RISK-PCI).

**Methods:**

This single-center retrospective study involved 200 patients with acute coronary syndrome (ACS) who underwent invasive diagnostic approach, ie, coronary angiography and myocardial revascularization if appropriate, in the period from January 2014 to July 2014. The GRACE, TIMI, and RISK-PCI risk scores were compared for their predictive ability. The primary endpoint was a composite 30-day major adverse cardiovascular event (MACE), which included death, urgent target-vessel revascularization (TVR), stroke, and non-fatal recurrent myocardial infarction (REMI).

**Results:**

The c-statistics of the tested scores for 30-day MACE or area under the receiver operating characteristic curve (AUC) with confidence intervals (CI) were as follows: RISK-PCI (AUC = 0.94; 95% CI 1.790-4.353), the GRACE score on admission (AUC = 0.73; 95% CI 1.013-1.045), the GRACE score on discharge (AUC = 0.65; 95% CI 0.999-1.033). The RISK-PCI score was the only score that could predict TVR (AUC = 0.91; 95% CI 1.392-2.882). The RISK-PCI scoring system showed an excellent discriminative potential for 30-day death (AUC = 0.96; 95% CI 1.339-3.548) in comparison with the GRACE scores on admission (AUC = 0.88; 95% CI 1.018-1.072) and on discharge (AUC = 0.78; 95% CI 1.000-1.058).

**Conclusions:**

In comparison with the GRACE and TIMI scores, RISK-PCI score showed a non-inferior ability to predict 30-day MACE and death in ACS patients. Moreover, RISK-PCI was the only scoring system that could predict recurrent ischemia requiring TVR.

Variations in clinical appearance and outcome in patients with acute coronary syndrome (ACS) require a reliable method of risk assessment for further major adverse cardiac events (MACE) during the ACS treatment. Risk stratification is a useful tool for planning early treatment, discharge, and rehabilitation of ASC patients, performing research after ACS, and expediting the definitive decision about further treatment (1,2).

Different scores are in use for the estimation of ACS outcome. Thrombolysis in myocardial infarction (TIMI) is a simple bedside score that predicts 30-day mortality in patients with ST-elevation myocardial infarction (STEMI). Also, the TIMI score evaluates 14-day adverse events in patients with unstable angina (UA)/non-ST-segment elevation myocardial infarction (NSTEMI). Several studies confirmed the efficacy of this index in the prognosis of mortality within 30-day period, although some attenuation was shown for patients older than 65 years (3-6). However, the TIMI score was based on clinical studies including selected populations of patients with low comorbidity rates and it does not always reflect the reality in clinical practice.

Global registry for acute coronary events (GRACE) score demonstrated its superiority in the assessment of 6-months mortality risk (7,8). Unlike TIMI, the GRACE score was derived from the Global Registry of Acute Coronary Syndrome data. As it was based on a wider sample of unselected population (9), it is more reliable for the routine clinical use. Several international reports compared the GRACE score with the TIMI score, showing that the GRACE score was a better predictor of clinical outcome than the TIMI score (10,11).

Predicting 30-day major adverse cardiovascular events after primary percutaneous coronary intervention (RISK-PCI score) was developed to predict 30-day MACE, such as non-fatal REMI, death, and cerebrovascular insult, in patients with STEMI after percutaneous coronary intervention (PCI) (12). The definitive value of all the scores is influenced by various clinical factors, such as score variables, period of the score evaluation, patient population, and analyzed adverse cardiovascular outcomes. RISK-PCI score has not been tested in patients with UA/NSTEMI. Although the GRACE score has already been established as the score of choice for the risk estimation (13), RISK-PCI also seems to be reliable considering that it includes clinical, laboratory, and diagnostic parameters. Our hypothesis was that the RISK-PCI score was as accurate as the GRACE and TIMI scores in predicting a composite MACE in patients with UA/NSTEMI.

The aim of our study was to estimate the prognostic potential of the RISK-PCI score and compare it with GRACE and TIMI scores for prediction of composite MACE occurrence within 30 days of a patient’s presentation with ACS.

## METHODS

### Patients

This single-center retrospective study included 200 patients with signs and symptoms compatible with ACS who were admitted to hospital between January 2014 and July 2014. There were 140 men and 60 women, with the mean age (±standard deviation, SD) of 62.3 ± 10.8 years (Table 1). The signs and symptoms compatible with ACS were acute chest pain or equivalent, and/or increase of cardiac troponin over the upper reference limit, and/or new significant ST- segment–T wave (ST–T) changes or new left bundle branch block [LBBB] in electrocardiogram (ECG), and/or images showing loss of viable myocardium or regional contractile abnormality (14). Clinical data, including previous medical history and risk factors, were taken into consideration.

**Table 1:** Baseline demographic, clinical, angiographic, and procedural characteristics of the patients with acute coronary syndrome*

	No. (%) of patients		
Patient characteristics	ACS total (N = 200)	NSTEMI (n = 69) and UA (n = 15)	STEMI (n = 116)
Age (years, mean±SD)	62.3 ± 10.8	63.3 ± 10.4	61.4 ± 11.1
Sex (men/women)	140/60	55/29	85/31
Medical history			
prior MI	40 (20.0)	25 (29.8)	15 (12.9)
prior AP	151 (75.5)	76 (90.5)	75 (64.6)
prior CHF	39 (19.5)	26 (31.0)	13 (11.2)
anterior MI	57 (28.5)	23 (27.4)	34 (29.3)
dyslipidemia	174 (87.0)	74 (88.1)	100 (86.2)
>3 standard risk factors	140 (70.0)	66 (78.6)	74 (63.8)
hypertension	130 (65.0)	63 (75.0)	67 (57.8)
diabetes mellitus	26 (13.0)	10 (11.9)	16 (13.8)
current smokers	112 (56.0)	41 (48.8)	70 (60.3)
Clinical characteristics			
KILLIP^†^ class I	156 (78.0)	62 (73.8)	94 (81.0)
KILLIP^†^ class II	41 (20.5)	20 (23.8)	21 (18.1)
KILLIP^†^ class III	3 (1.5)	2 (2.4)	1 (0.9)
asystoly	5 (2.5)	0 (0.0)	5 (4.3)
ST elevation	116 (58.0)	0 (0.0)	116 (100.0)
ST depression	117 (58.5)	55 (65.5)	62 (53.4)
AVB III	4 (2.0)	0 (0.0)	4 (3.4)
ABBB	10 (5.0)	3 (3.6)	7 (6.0)
heart rate (beats/min; median with interquartile range)	80.0 (40.0-300.0)	80.0 (47.0-120.0)	78.5 (40.0-300.0)
SBP (mmHg, median with interquartile range)	140.0 (50.0-220.0)	150.0 (90.0-220.0)	135.0 (50.0-220.0)
LVEF (%, median with interquartile range)	45.0 (5.0-66.0)	45.0 (15.0-65.0)	45.0 (5.0-66.0)
Laboratory data on admission			
leukocyte count (x 10^−9^/L; median with interquartile range)	9.9 (4.4-28.9)	9.5 (4.4-22.5)	10.4 (4.7-28.9)
glucose (mmol/L, median with interquartile range)	7.7 (3.6-53.6)	7.2 (4.1-23.0)	8.2 (3.6-53.6)
creatinine (mmol/L, median with interquartile range)	104.0 (56.4-148.8)	104.5 (70.1-152.0)	103.9 (56.4-1488.0)
creatinine clearance (mL/min, median with interquartile range)	69.9 (29.8-171.9)	66.0 (29.8-138.3)	71.8 (30.9-171.9)
peak troponin I (µg/L; median with interquartile range)	0.8 (0.0-50.0)	0.5 (0.0-11.2)	1.6 (0.0-50.0)
Angiographic findings			
diameter of IRA≤2,5 mm (median with interquartile range)	24 (12.0)	11 (13.1)	13 (11.2)
pre-procedural TIMI flow 0	78 (39.0)	13 (15.5)	65 (56.0)
final TIMI flow grade <3	63 (31.5)	32 (38.1)	31 (26.7)
temporary pacemaker	4 (2.0)	0 (0.0)	4 (3.4)

*Abbreviations: ACS – acute coronary syndrome; NSTEMI – non ST elevation myocardial infartion; UA – unstable angina; STEMI – ST elevation myocardial infarction; SD – standard deviation; MI – myocardial infarction; AP – angina pectoris; CHF – congestive heart failure; AVB III – atrioventricular block grade III; ABBB – acute bundle branch block; SBP – systolic blood pressure; LVEF – left ventricular ejection fraction; IRA – infarction-related artery; CABG – coronary artery bypass grafting.

†KILLIP – classification of the severity of heart failure by Thomas Killip: class I – no heart failure; class II - heart failure, diagnostic criteria include rales, S3 gallop, and venous hypertension; class III - pulmonary edema; class IV – cardiogenic shock.

Exclusion criteria were refusal to give consent for invasive treatment, active or recent internal bleeding, history of bleeding after non-steroid anti-inflammatory agents, known bleeding diathesis, intracerebral mass or aneurysm, intolerance or allergy to acetylsalicylates or clopidogrel, history of hypersensitivity to iodinated contrast media, cardiogenic shock at admission, non-cardiac conditions that could interfere with compliance with the protocol or necessitate interruption of the treatment with thienopyridines, and coexisting conditions associated with a limited life expectancy at 30-day follow-up. Revascularization procedure was performed in all patients in accordance with the applicable clinical practice guidelines for PCI (15). Before PCI, patients had been administered anticoagulant therapy and a loading dose of acetylsalicylates and clopidogrel. All patients were informed in detail about the intervention procedure and all signed a written consent.

Primary PCI was performed via femoral approach, using standard 6F or 7F guiding catheters. The procedure began with a visualization of the non-infarction related artery (IRA), followed by the visualization of the IRA. Stenting was performed in patients with anatomically-suitable artery with a diameter of ≥2.0 mm and without excessive tortuosity and extremely angulated lesions. Angioplasty without stenting was performed on selected patients who were candidates for urgent surgery or patients with small and tortuous or highly calcified IRA. Drug-eluting stents (DES) were suggested for selected patients with in-stent restenosis, diabetes mellitus, very long lesions, or bifurcated lesions. The decision about DES implantation was left to the discretion of the surgeon. In our trial, manual aspiration was used sporadically and was not included in the final analysis. Flow grades were assessed according to the TIMI criteria. Temporary pacemaker was placed in all patients with high-grade atrioventricular (AV) block or bradiarrhythmia and hemodynamic compromise.

After PCI, dual antiplatelet therapy was prescribed for 12 months to all patients included in the study.

### Method

The primary endpoint was a composite 30-day MACE consisting of death, target-vessel revascularization (TVR), stroke, and non-fatal recurrent myocardial infarction (REMI). TVR was defined as ischemia-driven revascularization of the IRA during a follow-up period (16). Stroke was defined as a new onset of focal or global neurological deficit lasting more than 24 hours. The presence of recurrent ischemic symptoms for 20 minutes or longer, reoccurrence of ST-segment deflection, T wave inversion or new pathognomonic Q waves in at least two contiguous leads, and increase in cardiac troponin above the upper reference limit and over 20% of the lowest recovery level from the index myocardial infarction (MI) were considered as REMI (14).

A planned staged PCI was not considered a TVR. All patients were followed up for 30 days or until death, whichever occurred first. Follow-up data were obtained in scheduled telephone interviews and outpatient visits. No patient was lost to follow-up.

Four scores computed from the previously acquired data included TIMI score, GRACE score on admission, GRACE score on discharge, and RISK-PCI score.

The TIMI score for NSTEMI and UA involves patient age, risk factors for coronary artery disease (hypertension, dyslipidemia, diabetes mellitus, active smoking, and heredity for coronary disease), prior coronary stenosis of 50% or more, use of acetylsalicylates in the previous seven days, at least 2 angina events in the previous 24 hours, increased serum cardiac biomarkers, and ST-segment deviation on ECG at presentation. The score of seven points is an important predictor of the development of adverse events such as new MI or REMI, death or severe recurrent ischemia requiring urgent revascularization (17). The TIMI score for STEMI involves patient age, systolic blood pressure value, heart failure, body mass, anterior MI or left bundle branch block, presence of diabetes mellitus, hypertension or angina pectoris, and the period between the symptom onset and the beginning of treatment (18).

The GRACE score on admission includes patient age, heart rate, systolic blood pressure value, increased laboratory cardiac markers and serum creatinine level, and ST deviation or asystole on ECG (7). The GRACE score on discharge includes patient age, aforementioned clinical signs and laboratory parameters, ST depression on ECG, prior MI, and in-hospital coronary intervention (19). The GRACE ACS risk calculators are available online (20).

The RISK-PCI score includes 12 variables as follows: patient age, prior MI, anterior MI, acute bundle branch block and high-grade AV block, laboratory findings (leukocyte counts, glycemia, and creatinine clearance), echocardiographic evaluation of left ventricular ejection fraction (EF), angiographic assessment of the IRA diameter, and initial and post-procedural TIMI flow grade. The total score ranges from 0 to 20. Patients with 5-6.5 score are at high risk (12).

### Statistical analysis

Statistical analysis was performed using a commercially available statistical software program (SPSS 13.0, Inc, Chicago Il, US). Normality of data distribution was assessed using Smirnov- Kolmogorov test. In addition to the usual parameters of central tendency (mean with standard deviation [SD]), Student’s *t* test was applied to test for differences between two related samples. For the non-parametric data, analysis was performed using the Mann-Whitney test, receiver operating characteristic (ROC) curve, and univariate and multivariate logistic regression analysis. The discrimination of the model was analyzed by calculating the area under the curve (AUC), which represented the accuracy of each value in discriminating high from low risk for patients with adverse events. AUC≥0.90 was defined as ‘excellent’, AUC≥0.70 was ‘good’, and AUC<0.70 was considered ‘inadequate’ (21). Calibration was evaluated by the Hosmer-Lemeshow goodness-of-fit test. The probability level in all tests was set at 95%.

## RESULTS

**Figure 1:**
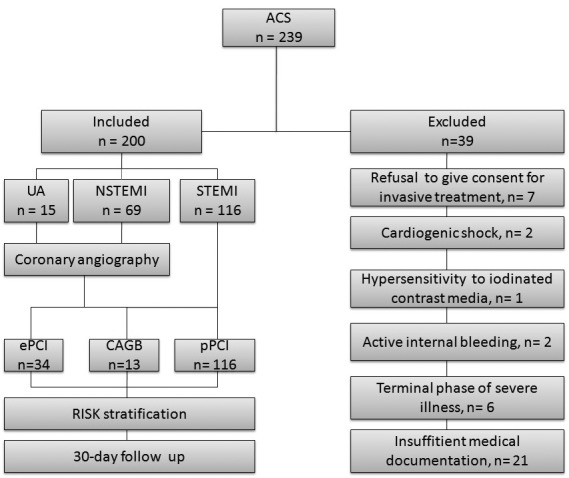
Flow diagram of the patients. ACS – acute coronary syndrome; UA – unstable angina; NSTEMI – non ST elevation myocardial infarction; STEMI – ST elevation myocardial infarction; ePCI – elective percutaneous coronary intervention; pPCI – primary percutaneous coronary intervention; CAGB – coronary arterial bypass grafting.

Of 200 ACS patients included in the study, STEMI was diagnosed in 116 (58.0%), NSTEMI in 69 (34.5%), and UA in 15 (7.5%) patients (Figure 1).

### Incidence of clinical events

The primary composite endpoint MACE occurred in 17 patients (8.5%) during the 30-day follow-up period including death in six cases (3.0%), REMI in two cases (1.0%), and urgent myocardial revascularizations due to current ischemia in 10 cases (5.2%). One patient had two undesired effects, reinfarction and subsequent fatal outcome. No stroke occurred during the follow-up period. Of the total of 200 patients, 122 (61.0%) underwent PCI and 13 (6.5%) underwent coronary artery bypass grafting (CAGB) in the same period. Four patients (2%) died in the hospital. The cause of death of three patients was the progression of heart failure and one patient had a fatal REMI. None of the patients used mechanical circulatory assistance while hospitalized. Two patients died after discharge from hospital within 20 days.

### Risk model calibration and discrimination

**Figure 2:**
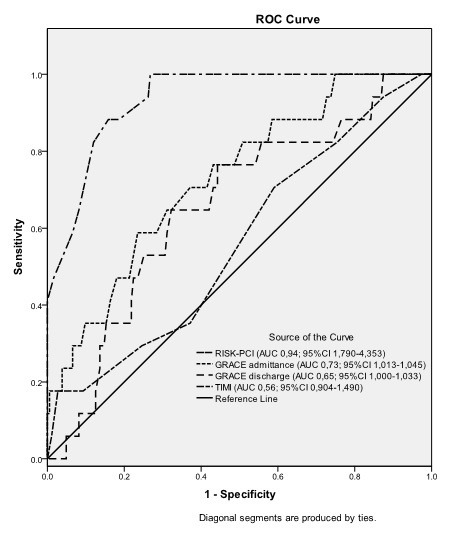
Receiver-operating characteristic (ROC) curves showing the discriminative ability of the risk scores for the prediction of 30-day composite major adverse cardiovascular events.

**Figure 3:**
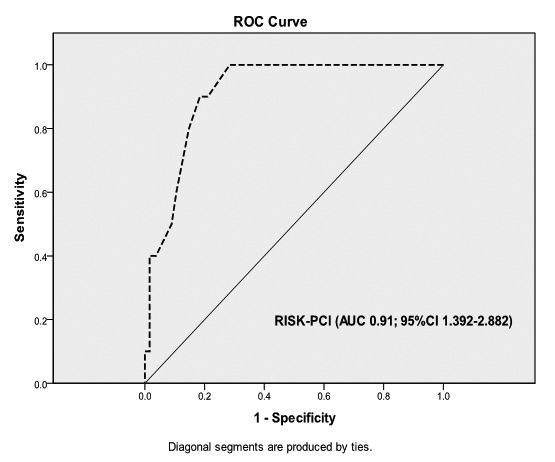
Receiver-operating characteristic (ROC) curves showing the discriminative ability of the RISK-PCI algorithm, the score predicting 30-day major adverse cardiovascular events after primary percutaneous coronary intervention, for the prediction of 30-day target-vessel revascularization (TVR).

**Figure 4:**
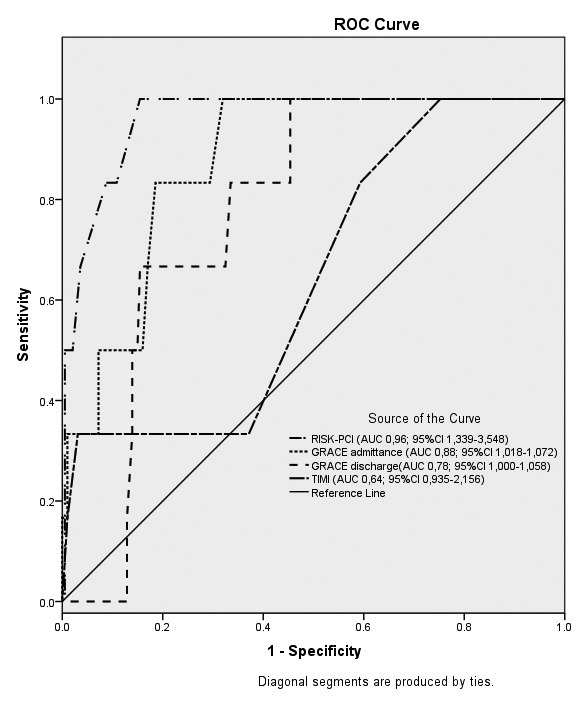
Receiver-operating characteristics (ROC) curves showing the discriminative ability of the risk scores for the prediction of 30-day death.

The RISK-PCI score expressed an excellent predictive potential for 30-day MACE (AUC = 0.94; 95% CI 1.790-4.353; *P* < 0.001) in comparison with the GRACE scores on admission (AUC = 0.73; 95% CI 1.013-1.045; *P* = 0.002) and discharge (AUC = 0.65; 95% CI 1.000-1.033; *P* = 0.035; Figure 2). The RISK-PCI score was the only score that could point toward TVR (AUC = 0.91; 95% CI 1.392-2.882; *P* < 0.001; Figure 3). The RISK-PCI score and GRACE scores on admission and discharge showed good discrimination performance for death within 30 days of admission. The RISK-PCI score demonstrated an excellent discriminative potential for 30-day death (AUC = 0.96; 95% CI 1.339-3.548; *P* < 0.001) in comparison with the GRACE score on admission (AUC = 0.88; 95% CI 1.018-1.072; *P* = 0.002) and discharge (AUC = 0.78; 95% CI 1.000-1.058; *P* = 0.021; Figure 4).

The TIMI score did not show any statistical significance in 30-day prediction of death (AUC = 0.64; 95% CI 0.935-2.156; *P* > 0.05), repeated urgent myocardial revascularization (AUC = 0.49; 95% CI 0.728-1.382; *P* > 0.05) or composite MACE (AUC = 0.56; 95% CI 0.904-1.490; *P* > 0.05).

The Hosmer-Lemeshow *P* values for all subgroups were >0.05, suggesting a good model fit (Table 2).

**Table 2:** Hosmer-Lemeshow calibration statistics for RISK-PCI, GRACE, and TIMI risk scores in patients with acute coronary syndrome*

Risk score	Death		TVR		MACE	
	χ^2^	*P*	χ^2^	*P*	χ^2^	*P*
RISK-PCI	0.851	0.997	3.509	0.834	2.527	0.922
GRACE on admission	2.156	0.976	4.319	0.827	4.540	0.805
GRACE on discharge	13.796	0.087	5.786	0.671	5.990	0.648
TIMI	6.605	0.252	2.499	0.777	3.046	0.693

*Abbreviations: TVR – target-vessel revascularization; MACE – major adverse cardiac events; RISK-PCI – predicting 30-day major adverse cardiovascular events after primary percutaneous coronary intervention; GRACE – Global Registry for Acute Coronary Events; TIMI – thrombolysis in myocardial infarction.

The logistic regression showed that the RISK-PCI score was an important predictor of death, urgent revascularization, and composite MACE, whereas the GRACE score on admission was adequate for predicting death and composite MACE.

The TIMI score was not found to have significant predictive potential for 30-day MACE (Table 3).

**Table 3:** Results of the univariable and multivariable logistic regression analyses for the RISK-PCI, GRACE, and TIMI scoring models*

Risk score	Death		TVR		MACE		MACE	
	univariate		univariate		univariate		multivariate	
	*P*	RR (95% CI for RR)	*P*	RR (95% CI for RR)	*P*	RR (95% CI for RR)	*P*	RR (95% CI for RR)
RISK PCI	0.002	2.180 (1.339-3.548)	<0.001	2.003 (1.392-2.882)	<0.001	2.791 (1.790-4.353)	<0.001	2.422 (1.429-3.519)
GRACE on admission	0.001	1.045 (1.018-1.072)	0.395	1.008 (0.989-1.028)	<0.001	1.029 (1.013-1.045)	/	/
GRACE on discharge	0.047	1.029 (1.000-1.058)	0.538	1.006 (0.986-1.027)	0.058	1.016 (0.999-1.033)	/	/
TIMI	0.100	1.420 (0.935-2.156)	0.987	1.003 (0.728-1.382)	0.243	1.160 (0.904-1.490)	/	/

*Abbreviations: TVR – target-vessel revascularization; MACE – major adverse cardiac events; RISK-PCI – the score predicting 30-day major adverse cardiovascular events after primary percutaneous coronary intervention; GRACE - Global Registry for Acute Coronary Events; TIMI – thrombolysis in myocardial infarction; CI – confidence intervals; RR – relative risk.

## DISCUSSION

This study was the first to compare different clinical scores, GRACE, TIMI, and newly introduced RISK-PCI, for their ability to predict possible adverse cardiovascular outcome in 30-day follow-up period (2,7,12,16). The RISK-PCI score reliably predicted a 30-day MACE in ACS patients and was non-inferior to the other two clinical scores. The most important advantage of RISK–PCI score was a prediction of TVR.

According to our results, the TIMI score was found to have the worst predictive potential, probably due to a relatively small number of parameters used, ie, symptom of the acute renal failure, generally accepted as an independent predictor of the poor in-hospital survival, was not considered at all (22,23). Nevertheless, the TIMI score seemed to be useful for a quick evaluation of ACS patients during the emergency department visit (24). The GRACE risk score had the highest prognostic accuracy for long-term mortality at each observed time point. The GRACE risk score is unique, because it is based on a large registry of patients across the entire spectrum of coronary syndromes and designed for the prognosis of all-cause mortality at six months (19,25). Furthermore, the GRACE score seems to be the most suitable for the prediction over a longer follow-up period (25). In our study, the RISK-PCI and GRACE scores on admission and discharge showed a good discriminative potential regarding the patient stratification into low- and high-risk groups. These two scoring systems also showed a good prognostic value for the adverse cardiovascular events in 30-day follow-up period. Specifically, every third patient with the RISK-PCI score ≥7 (very high-risk group) died during the 30-day follow-up, which implies that such patients need close monitoring during the acute phase of MI and careful evaluation of the dual antiplatelet therapy. Moreover, the high-risk patients might also be candidates for complete revascularization before discharge from hospital (12). The RISK-PCI score was found to be the most precise for the prediction of major cardiovascular adverse events, such as death or relevant ischemia requiring urgent revascularization. The RISK-PCI scoring system had the best predictive value for the MACE estimation, with a good discriminative ability, which is not surprising considering that RISK-PCI was designed for the MACE anticipation from the beginning. On the other hand, the GRACE scoring system was not initially intended for the MACE estimation, but it has been gradually used for the prediction of other adverse incidents during in-hospital stay, such as re-infarction, arrhythmias, acute congestive heart failure, shock, and cerebrovascular incidents (26,27). Prognostic information based exclusively on the ECG changes and cardiac markers is insufficient, because ECG changes have high specificity but low sensitivity for the myocardial ischemia. Furthermore, prognostic potential of the cardiac markers is attenuated due to the period of latency between the moment of cardiac myocyte necrosis and subsequent entrance of the markers into the circulation. Therefore, in the emergency department, risk indices are more favorable for the prognosis in comparison with ECG findings and troponin concentration (11,28). In recent studies, acute branch block (29,30), post interventional TIMI flow through the IRA (31,32), and left ventricle ejection fraction (31,33) were found prognostically important for long-term outcome. Furthermore, an anterior wall MI was associated with a significantly higher risk of mortality than an inferior wall MI. Renal insufficiency and age were emphasized as good MACE predictors (32-36). All these variables are included in the RISK-PCI score and this fact probably increases its predictive accuracy. Leukocyte count on admission makes the crucial difference between RISK-PCI and other two scoring systems, because its increased value positively correlates with lower subepicardial flow, frequent left chamber remodeling, congestive heart failure, and fatal outcome (37). Angiographic parameters are omitted from the GRACE score on admission and the TIMI score, while GRACE score on discharge takes into consideration only two similar variables, prior MI and eventual PCI performance (8). In the RISK-PCI score, angiographic assessment of the diameter of the infraction-related coronary artery and its TIMI flow are considered as the factor for the overall evaluation of the patient’s condition and subsequent selection on the basis of the seriousness of the symptoms. We found the RISK-PCI score to be an independent predictor of death, urgent revascularization, and composite MACE, and the GRACE score on admission to be an independent predictor of the fatal outcome after the 30-day follow-up period.

The main limitation of our study lies in its retrospective and single-center-based design. In consistency with the widely accepted risk models for primary PCI, patients with cardiogenic shock at presentation were excluded from the trial. These patients by definition fall into the highest-risk category and their treatment differs from the overall primary PCI population in terms of the time window for revascularization (no time limit) and the extent of revascularization (complete revascularization recommended). We performed only femoral approach in the aforementioned period. Therefore, our results do not refer to the radial approach.

Given a relatively small number of patients in our study, the results of the study should be considered as hypothesis-generating and further verified in a larger trial in future.

In conclusion, RISK-PCI showed a reasonably fair discriminative ability for the 30-day MACE in ACS patients. In comparison with the GRACE and TIMI scores, the RISK-PCI score showed a non-inferior ability to predict 30-day MACE and death and was the only index that could predict recurrent ischemia requiring TVR. Further research in larger patient cohorts is needed for the clinical validation of RISK-PCI score in patients with UA/NSTEMI.
